# CCL2 as a potential therapeutic target for clear cell renal cell carcinoma

**DOI:** 10.1002/cam4.886

**Published:** 2016-09-26

**Authors:** Ryuichiro Arakaki, Toshinari Yamasaki, Toru Kanno, Noboru Shibasaki, Hiromasa Sakamoto, Noriaki Utsunomiya, Takayuki Sumiyoshi, Shinsuke Shibuya, Tatsuaki Tsuruyama, Eijiro Nakamura, Osamu Ogawa, Tomomi Kamba

**Affiliations:** ^1^ Department of UrologyKyoto University Graduate School of MedicineKyotoJapan; ^2^ Department of Diagnostic PathologyKyoto University Graduate School of MedicineKyotoJapan; ^3^ Laboratory for Malignancy Control Research/Medical Innovation CenterKyoto University Graduate School of MedicineKyotoJapan

**Keywords:** Angiogenesis, chemokine (C‐C motif) ligand‐2, renal cell carcinoma, tumor macrophage, xenograft

## Abstract

We previously reported that the pVHL‐atypical PKC‐JunB pathway contributed to promotion of cell invasiveness and angiogenesis in clear cell renal cell carcinoma (ccRCC), and we detected chemokine (C‐C motif) ligand‐2 (CCL2) as one of downstream effectors of JunB. CCL2 plays a critical role in tumorigenesis in other types of cancer, but its role in ccRCC remains unclear. In this study, we investigated the roles and therapeutic potential of CCL2 in ccRCC. Immunohistochemical analysis of CCL2 expression for ccRCC specimens showed that upregulation of CCL2 expression correlated with clinical stage, overall survival, and macrophage infiltration. For functional analysis of CCL2 in ccRCC cells, we generated subclones of WT8 cells that overexpressed CCL2 and subclones 786‐O cells in which CCL2 expression was knocked down. Although CCL2 expression did not affect cell proliferation in vitro, CCL2 overexpression enhanced and CCL2 knockdown suppressed tumor growth, angiogenesis, and macrophage infiltration in vivo. We then depleted macrophages from tumor xenografts by administration of clodronate liposomes to confirm the role of macrophages in ccRCC. Depletion of macrophages suppressed tumor growth and angiogenesis. To examine the effect of inhibiting CCL2 activity in ccRCC, we administered CCL2 neutralizing antibody to primary RCC xenografts established from patient surgical specimens. Inhibition of CCL2 activity resulted in significant suppression of tumor growth, angiogenesis, and macrophage infiltration. These results suggest that CCL2 is involved in angiogenesis and macrophage infiltration in ccRCC, and that CCL2 could be a potential therapeutic target for ccRCC.

## Introduction

Kidney cancer, or renal cell carcinoma (RCC), accounts for about 4% of all adult malignancies in the United States 1. Clear cell renal cell carcinoma (ccRCC) is the most common histological subtype of RCC and represents approximately 85% of RCC 2. Mutation of the von Hippel–Lindau tumor suppressor gene (VHL) occurs in approximately 60% of sporadic ccRCCs 3. The identification of a dysregulated pVHL‐hypoxia inducible factor (HIF) pathway in ccRCC has led to a new treatment paradigm in targeted therapeutic approaches. Molecularly targeted agents that target vascular endothelial growth factor (VEGF), VEGF receptor (VEGFR), and the mammalian target of rapamycin complex 1 (mTORC1) have been developed and used in clinical practice, and are more effective than previous immunotherapeutic agents 4. Although many patients with advanced RCC experience clinical benefit from these treatments, these drugs generally only slow the progression of disease and ultimately result in disease relapse 5. Therefore, the identification of new therapeutic targets in advanced RCC is necessary.

We previously reported that the pVHL‐atypical PKC‐JunB pathway contributed to promote cell invasiveness and enhance angiogenesis in ccRCC in a HIF‐independent manner 6. Furthermore, microarray analysis revealed chemokine (C‐C motif) ligand‐2 (CCL2) was one of downstream effectors of JunB 6. CCL2, also referred to as monocyte chemoattractant protein‐1 (MCP1), is the first discovered human CC chemokine and one of the key chemokines that regulate migration and infiltration of monocytes/macrophages 7. CCL2 also plays a critical role in promoting tumorigenesis through the recruitment of macrophages and the induction of angiogenesis in other types of cancer 8, 9. Previous studies showed that intratumoral infiltration of tumor‐associated macrophages (TAMs) promotes tumor progression directly or indirectly 10, 11. Therefore, the aim of this study was to investigate the roles of CCL2 and explore its therapeutic potential in ccRCC.

## Materials and Methods

### Patients and RCC samples

Tumor specimens from 114 patients who received radical or partial nephrectomy with appropriate informed consent were obtained at the Department of Urology at Kyoto University Hospital under the protocols approved by the University's institutional review board (IRB approval number G52).

### Cell culture

The 786‐O cell line was purchased from the American Type Culture Collection (Rockville, MD). The 786‐O subclones stably transfected with pRc‐CMV (pRC3) or pRc‐CMV‐HA‐VHL (WT8) 12, and UMRC2 cells and UMRC2 subclones stably transfected with pLenti6‐HA (UMRC2‐HA) or pLenti6‐HA‐LHVHL (UMRC2‐VHL) were a kind gift from Dr. William G. Kaelin (Dana‐Farber Cancer Institute, Boston, MA). The cells were cultured as previously described 13, 14. Cells (786‐O cells and UMRC2) stably infected with shRNA lentivirus and stably infected with pBABE‐puro retroviruses were selected in the presence of 1.5 *μ*g/mL puromycin.

### Cell proliferation assays

We seeded 1000 cells into 96‐well plates in DMEM with 10% FBS. Proliferative activity was determined by the MTT assay using a microtiter plate reader at 540 nm. All experiments were performed in triplicate.

### Mouse xenograft assays

All experiments involving laboratory animals were performed in accordance with the Guidelines for Animal Experiments of Kyoto University. To establish primary xenograft models, local tumors of the kidney were resected by radical nephrectomy, minced into 20–30 mm^3^ fragments, and subcutaneously transplanted into 5‐week‐old CB‐17/Icr‐crj SCID mice (Charles River, Yokohama, Japan) on the day of surgery. Xenografts were established within 4 months after the first inoculation. Xenograft tumors were extracted and transplanted into several SCID mice.

To establish cell line xenograft models, a total of 1.0 × 10^7^ cells were injected subcutaneously into the unilateral flank of 5–6‐week‐old female BALB/cA Jcl nude (nu/nu) mice (CLEA, Tokyo, Japan). Tumor volumes were measured twice a week. All animals were euthanized and tumors were excised.

### CCL2 ELISA

CCL2 protein levels in the supernatant of cell culture or tissues of xenografts were quantified using CCL2 ELISA kits (R&D Systems, Minneapolis, MN). Concentration (pg/mL) was normalized to total cell or tissue protein (mg).

### Antibodies and reagents

Antibodies were purchased commercially as follows: mouse CD31 (clone MEC 13.3; BD Pharmingen, San Diego, CA), mouse F4/80 (clone BM8; eBioscience, San Diego, CA), human CD68 (clone PG‐M1; Dako, Glostrup, Denmark), human CCL2 (R&D Systems), mouse iNOS (#ab15323; abcam, Cambridge, MA), mouse arginase1 (#sc‐20150; Santa Cruz biotechnology, Santa Cruz, CA), and mouse MRC1/CD206 (#ab64693; abcam).

### Drugs and treatments

Neutralizing antibodies to human CCL2 (MAB679; R&D Systems) or control mouse IgG were given to mice with primary xenograft tumors by intraperitoneal injection twice a week at a dose of 10 *μ*g/mouse 15. The dose of bevacizumab was determined based on previous studies 16. Treatment with bevacizumab (100 *μ*g dissolved in 0.9% NaCl solution) or vehicle was started simultaneously in each cohort of xenografts at 4–5‐week intervals after tumor inoculation. Intraperitoneal injections were performed twice a week. Clodronate liposome (Hygieia Bioscience, Osaka, Japan) or control liposome was given to mice with subcutaneous tumors from WT8 mock or WT8/CCL2 cells by intravenous injection through the tail vein every 4 days at a dose of 100 *μ*l/mouse 17, 18.

### Immunohistochemistry and immunoquantification

**Figure 1:**
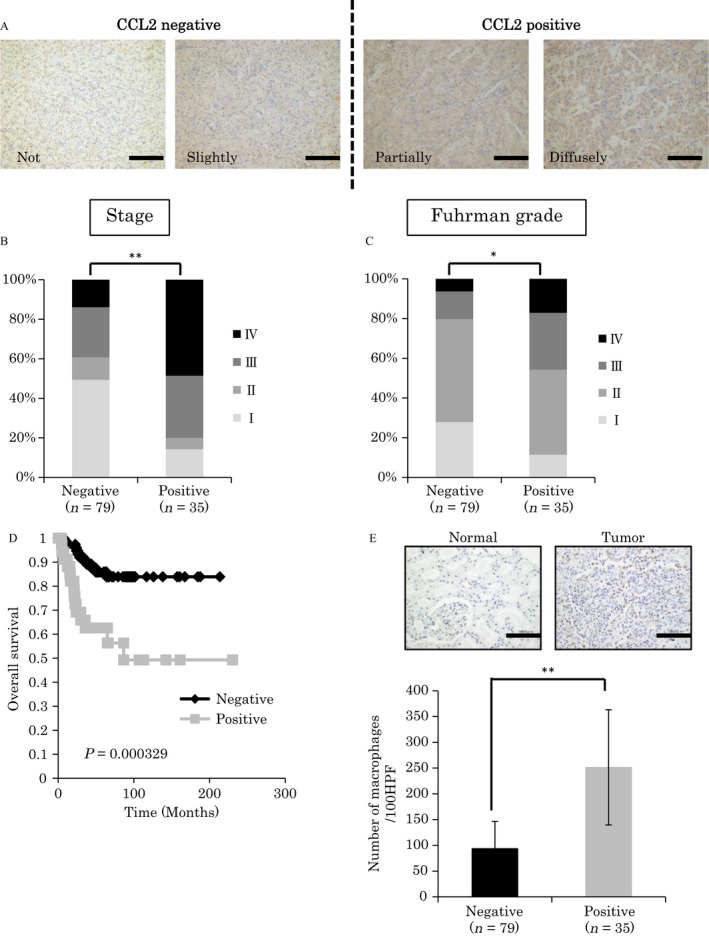
Chemokine (C‐C motif) ligand‐2 (CCL2) expression in clear cell renal cell carcinoma (ccRCC) clinical specimens. (A) Examples of ccRCC samples showing “not stained”, “slightly stained”, “partially stained”, and “diffusely stained” CCL2 staining (bar: 100 *μ*m). (B) Statistical analysis for clinical stage of patients in the CCL2‐negative group (“not stained” and “slightly stained”, *n* = 79) compared with patients in the CCL2‐positive group (“partially stained” and “diffusely stained”, *n* = 35). Statistical analysis was performed using Mann–Whitney nonparametric *U*‐test (**P <* *0.01). (C) Statistical analysis for Fuhrman grade of patients in the CCL2‐negative group (*n* = 79) compared with patients in the CCL2‐positive group (*n* = 35). Statistical analysis was performed using Mann–Whitney nonparametric *U*‐test (*P <* *0.05). (D) Overall survival rates of 114 ccRCC patients were determined according to CCL2 expression by Kaplan–Meier curves, with the event being defined as death related to cancer (79 patients in the CCL2‐negative group and 35 patients in the CCL2‐positive group). The log‐rank test was used to identify differences between the survival curves of different patient groups (*P*  = 0.000329). (E) Examples of CD68 staining in ccRCC samples (top panels, bar: 100 *μ*m). Statistical analysis of the number of recruited macrophages in tumors in the CCL2‐negative group (*n* = 79) and CCL2‐positive group (*n* = 35) (bottom table). Statistical analysis was performed using the Students' *t*‐test (**P <* *0.01).

Immunohistochemical analysis was carried out on formalin‐fixed, paraffin‐embedded clinical tissues or OCT compound (Tissue Tek; Sakura Finetek, Torrance, CA) fixed xenograft tissues. As CCL2 is a soluble form of protein, CCL2 protein levels in whole ccRCC lysate are optimally evaluated using ELISA. Considering the clinical implication, we applied immunohistochemistry to determine the CCL2 expression levels with formalin‐fixed samples in this study as previously described 6, 14. In human ccRCC tissue, CCL2 staining was detected in both tumor cells and stromal cells. The numbers of cells stained for CCL2 were counted and samples were categorized as “not stained (0–25%)”, “slightly stained (25–50%)”, “partially stained (50–75%)”, and “diffusely stained (75–100%)” by one pathologist without prior knowledge of clinical features (Fig. 1A). In this study, “partially stained” and “diffusely stained” were defined as positive, and “not stained” and “slightly stained” were defined as negative.

Tumor microvessel density (MVD) was evaluated using CD31 as the endothelial marker in three different areas at 100× magnification as suggested by Weidner 19. The macrophages were counted by counting the F4/80‐positive (mouse macrophage) or CD68‐positive cells (human macrophage) at 200× magnification in a manner similar to MVD. The mean count of three areas was calculated as MVD and the macrophage cell count.

### Protein extraction and immunoblot analysis

Whole‐cell proteins were isolated from cultured cells, followed by immunoblotting as previously described 13, 14.

### Real‐time PCR and PCR arrays

cDNA synthesis and real‐time PCR for CCL2 and GAPDH were performed as previously described 14, 20. The primer sequences were as follows: CCL2, 5′‐CCCCAGTCACCTGCTGTTAT‐3′ (sense) and 5′‐AGATCTCCTTGGCCACAATG‐3′ (antisense); and GAPDH, 5′‐GAAGGTGAAGGTCGGAGTC‐3′ (sense) and 5′‐GAAGATGGTGATGGGATTTC‐3′ (antisense).

### Plasmid construction and retroviral expression

Full‐length CCL2 cDNA was amplified by PCR from 786‐O and UMRC2 cell cDNA using PrimeSTAR HS DNA polymerase (Takara Bio, Shiga, Japan) and cloned into the pBABE‐puro retroviral vector. The oligonucleotide sequences used in the construction of the expression construct were as follows: *CCL2*, 5′‐CGCGGATCCATGAAAGTCTCTGCCGCCCTTCT‐3′ and 5′‐GACGTCGACTCAAGTCTTCGGAGTTTGGGTTT‐3′. The PCR products were inserted into pBABE‐puro vector at BamH1/Sal1 sites using an Infusion‐HD Cloning Kit (Takara Bio). G3T‐hi packaging cells were transfected with retroviral plasmids using a Retrovirus Packaging Kit (Ampho; Takara Bio). These experiments were performed according to the manufacturer's instructions.

### Statistical analysis

Data are expressed as the mean ± SD. Student's *t*‐test was used to analyze the differences between means. Mann–Whitney nonparametric *U*‐test was performed to compare differences in clinical stage between the CCL2‐negative group and CCL2‐positive group. Tumor growth in vivo was analyzed by two‐way repeated ANOVA. A *P*‐value smaller than 0.05 was considered statistically significant.

## Results

### CCL2 expression correlated with clinical stage, overall survival, and macrophage infiltration in ccRCC clinical specimens

We first performed immunohistochemical analysis of CCL2 expression in a total of 114 ccRCC specimens. The patients' characteristics are shown in Table 1. Higher CCL2 expression was associated with worse clinical stage and Fuhrman grade (Fig. 1A, B, and C). Patients in the CCL2‐positive group had a significantly lower overall survival compared with patients in the CCL2‐negative group (*P *=* *0.000329, log‐rank test, Fig. 1D). Because CCL2 is a chemoattractant for macrophages, we speculated whether CCL2 promotes macrophage recruitment in ccRCC clinical specimens. Interestingly, there was a trend between CCL2 expression and macrophage infiltration (Fig. 1E). Analysis of gene expression data in the Oncomine website showed that human primary ccRCC tissues express significantly higher levels of CCL2 mRNA compared with those of normal kidney tissues (Fig. S1) 21. These data suggest that CCL2 expression is associated with disease progression, tumor malignancy, and macrophage infiltration in ccRCC specimens.

**Table 1:** Clinical pathologic characteristics of clear cell renal cell carcinoma patients

Characteristics	No. of patients (*N* = 114)
Gender	
Male	72
Female	42
Age (year)	
Range	29–84
Median	63
Stage	
I	45
II	10
III	31
IV	28
Fuhrman grade	
I	26
II	56
III	21
IV	11

### CCL2 expression was also related to tumor growth, angiogenesis, and macrophage infiltration in vivo

**Figure 2:**
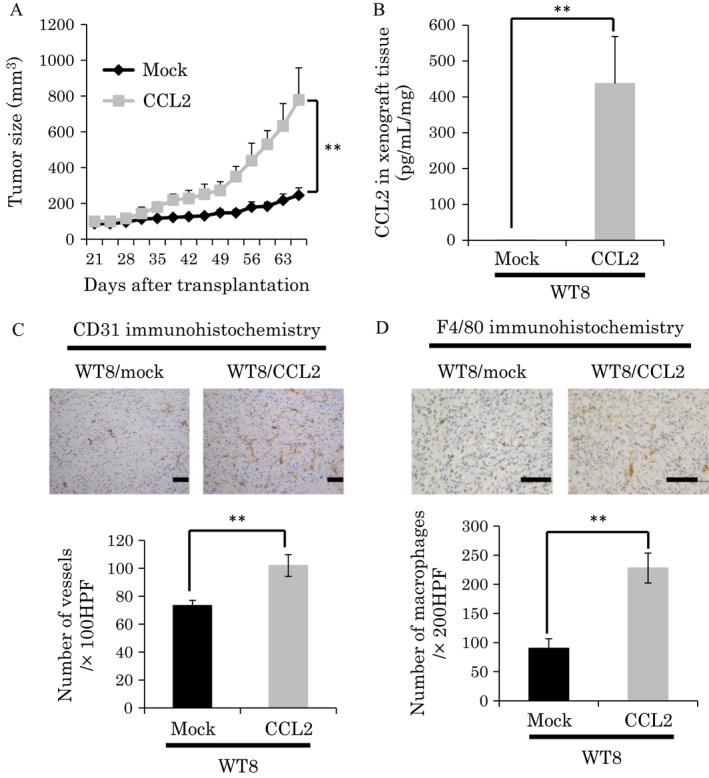
Tumor growth and immunohistochemistry analysis of chemokine (C‐C motif) ligand‐2 (CCL2) overexpressing WT8 cells. (A) Local tumor xenografts were established by subcutaneous injection of WT8 mock or WT8 CCL2 cells in nude mice (*n* = 4 mice per group), and tumor growth was monitored as described in Materials and Methods. Values represent mean ± SD. Statistical analysis was performed using two‐way repeated ANOVA (**P <* *0.01). (B) Expression of CCL2 was analyzed in tumor tissues from WT8 mock or WT8 CCL2 cells by ELISA. Values represent mean ± SD. Statistical analysis was performed using the Students' *t*‐test (**P <* *0.01). (C) Immunohistochemical detection of CD31 in tumor tissues from WT8 mock or WT8 CCL2 cells (top panels, bar: 100 *μ*m) and quantitative analysis of CD31 staining (bottom graph). Values represent mean ± SD. Statistical analysis was performed using the Student's *t*‐test (**P <* *0.01). (D) Immunohistochemical detection of F4/80 in tumor tissues from WT8 mock or WT8 CCL2 cells (top panels, bar: 100 *μ*m) and quantitative analysis of F4/80 staining (bottom graph). Values represent mean ± SD. Statistical analysis was performed using the Student's *t*‐test (**P <* *0.01).

**Figure 3:**
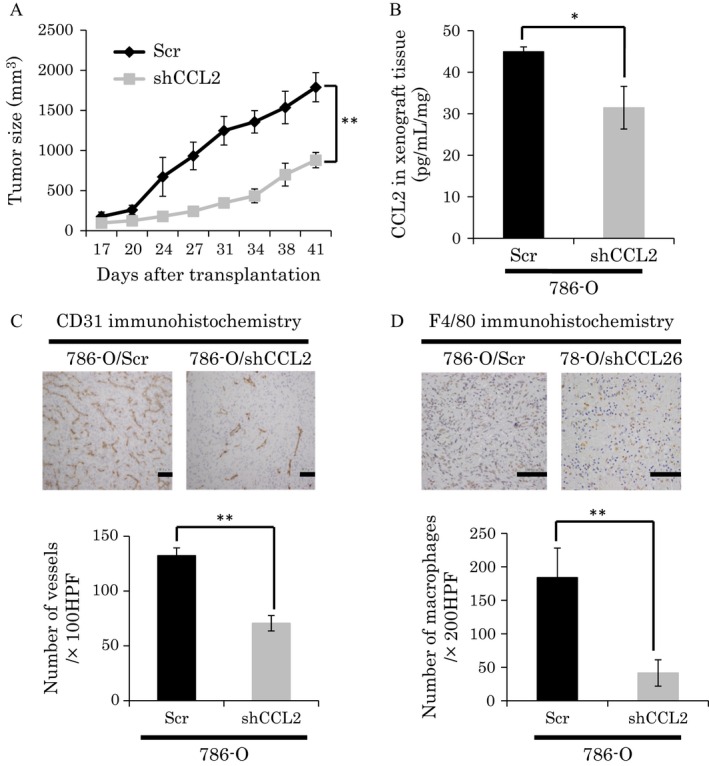
Tumor growth and immunohistochemistry analysis of chemokine (C‐C motif) ligand‐2 (CCL2) knockdown 786‐O cells. (A) Local tumor xenografts were established by subcutaneous injection of 786‐O scramble or 786‐O shCCL2 cells to nude mice (*n* = 4 mice per group), and tumor growth was monitored as described in Materials and Methods. Values represent mean ± SD. Statistical analysis was performed using two‐way repeated ANOVA (**P <* *0.01). (B) Expression of CCL2 was analyzed in tumor tissues of 786‐O scramble or 786‐O shCCL2 cells by ELISA. Values represent mean ± SD. Statistical analysis was performed using the Student's *t*‐test (*P <* *0.05). (C) Immunohistochemical detection of CD31 in tumor tissues from 786‐O scramble or 786‐O shCCL2 cells (top panels, bar: 100 *μ*m) and quantitative analysis of CD31 staining (bottom table). Values represent mean ± SD. Statistical analysis was performed using the Student's *t*‐test (**P <* *0.01). (D) Immunohistochemical detection of F4/80 in tumor tissues from 786‐O scramble or 786‐O shCCL2 cells (top panels, bar: 100 *μ*m) and quantitative analysis of F4/80 staining (bottom table). Values represent mean ± SD. Statistical analysis was performed using the Student's *t*‐test (**P <* *0.01).

We previously reported CCL2 mRNA and protein expression in ccRCC cell lines as determined by qRT‐PCR and ELISA 14. CCL2 expression was higher in *VHL* ^*−/−*^ pRC3 cells than wt‐VHL‐expressing WT8 cells. Similarly, CCL2 expression was also higher in *VHL* ^*−/−*^ UMRC2‐HA cells than wt‐VHL‐expressing UMRC2‐VHL cells 14. We first evaluated the function of CCL2 using ccRCC cell lines by generating subclones of WT8 cells that overexpressed CCL2 (clone WT8/CCL2) (Fig. S2A). However, overexpression of CCL2 did not affect cell proliferation in vitro (Fig. S2B). We next explored the roles of CCL2 in regulating tumor growth in vivo using nude mouse xenograft models. WT8/CCL2 and WT8 mock cells were subcutaneously implanted into mice and tumor growth was monitored. Remarkably, the average growth of tumors from WT8/CCL2 cells was significantly more rapid than that of tumors from control WT8 mock cells (Fig. 2A). We confirmed that CCL2 expression was increased in tumor tissues derived from WT8/CCL2 cells (Fig. 2B). We examined blood vessel formation and macrophage recruitment by staining for CD31 and F4/80 in the subcutaneous xenograft tumors. Significant differences were observed in microvessel density (MVD) and macrophage infiltration between tumors from WT8/CCL2 cells and WT8 mock cells (Fig. 2C and D). Next, to examine the contribution of CCL2 expression in ccRCC, we also generated 786‐O subclones that were stably knocked down for CCL2 expression using shRNA (clone 786‐O/shCCL2). We confirmed that the shRNA construct efficiently repressed CCL2 mRNA and protein levels in 786‐O cells (Fig. S2C). Although shRNA‐mediated suppression of CCL2 in 786‐O cells did not affect proliferation in vitro (Fig. S2D), the xenograft tumor growth was reduced compared with that from 786‐O scramble cells in vivo (Fig. 3A). We confirmed that CCL2 expression was decreased in tumor tissues derived from 786‐O/shCCL2 cells (Fig. 3B). Immunohistochemistry for CD31 and F4/80 revealed that tumors from 786‐O/shCCL2 cells showed significantly lower MVD and macrophage infiltration than tumors from 786‐O scramble cells (Fig. 3C and D). Similar results were observed in experiments using UMRC2 cells (Fig. S3 and S4). Collectively, these gain‐ and loss‐of‐function studies suggested that CCL2 might be involved in tumor growth, angiogenesis, and macrophage infiltration in ccRCC.

### Depletion of macrophages inhibited tumor growth in ccRCC

**Figure 4:**
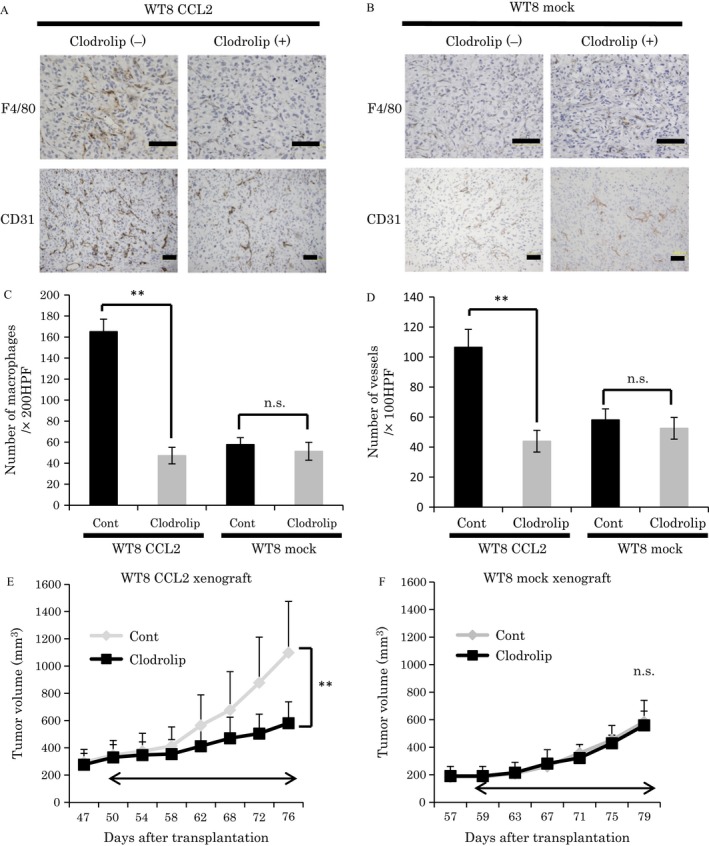
Effects of macrophage depletion on growth of clear cell renal cell carcinoma (ccRCC) tumors. Effect of macrophage depletion on the growth of tumor xenografts was investigated using liposome injection (*n* = 4 mice per each group). WT8 chemokine (C‐C motif) ligand‐2 (CCL2) or WT8 mock cells were injected subcutaneously into nude mice. Control liposomes or clodronate liposomes were given intravenously to the mice. (A, B) Images of F4/80 and CD31 immunohistochemical staining in the control liposome or clodronate liposome‐treated tumors from WT8 CCL2 (A) or WT8 mock cells (B) (bar: 100 *μ*m). (C, D) Quantification of macrophage infiltration (C) or microvessel density (MVD) (D) in control liposome or clodronate liposome‐treated tumors from WT8 CCL2 or WT8 mock cells. Values represent mean ± SD. Statistical analysis was performed using the Student's *t*‐test (**P <* *0.01, n.s.: not significant). (E, F) Tumor growth curve: tumor xenograft volume in the control liposome or clodronate liposome‐treated tumors from WT8 CCL2 (E) or WT8 mock cells (F). Values represent mean ± SD. Statistical analysis was performed using the two‐way repeated ANOVA (**P <* *0.01, n.s.: not significant).

To determine whether recruitment of macrophages plays a critical role in tumor growth in ccRCC, we depleted macrophages from tumor xenografts by administration of clodronate liposomes. Macrophages recognize liposomes as foreign bodies in vivo and undergo apoptosis by phagocytosis and release of the encapsulated clodronate 17. We established ccRCC xenografts from WT8/CCL2 cells in which macrophage infiltration was increased and treated them with clodronate liposome or control liposome. Immunohistochemical analysis showed a lower number of infiltrated macrophages in the clodronate liposome‐treated tumors compared with the control liposome‐treated tumors (Fig. 4A and C). Interestingly, depletion of macrophages suppressed not only tumor growth but also tumor angiogenesis (Fig. 4A, D, and E). On the other hand, in xenografts from WT8 mock cells in which macrophages infiltration was decreased, tumor growth and tumor angiogenesis remained unaffected by clodronate liposome treatment as well as control liposome treatment (Fig. 4B, D, and F). These results suggested that macrophages recruited by CCL2 could promote tumor growth in ccRCC.

### Inhibiting CCL2 with neutralizing antibody reduced tumor growth, MVD, and macrophage infiltration in RCC xenograft models

We next examined the effect of inhibiting CCL2 activity with a CCL2 neutralizing antibody (CCL2NA) in ccRCC. In xenograft tumors derived from CCL2‐secreting 786‐O cells, CCL2NA treatment suppressed tumor growth accompanied with a decreased MVD (Fig. S5A, C, and D), and these effects were comparable to those with bevacizumab treatment (Fig. S5B, C, and D). We observed significantly less macrophage infiltration in xenografts with CCL2NA treatment compared with those treated with control IgG or bevacizumab, indicating that macrophage recruitment was also sensitive to CCL2 inhibition (Fig. S5C and E). Unexpectedly, macrophage infiltration was significantly increased in tumors with bevacizumab treatment compared with those with IgG treatment.

We established several primary RCC xenografts from directly isolated patient surgical ccRCC specimens and they were stably engrafted following three or more passages in vivo 20. In this study, we used the primary RCC xenograft named KURC3 (Kyoto University Renal Cancer 3); the histopathological diagnosis of the primary tumor was ccRCC Fuhrman grade IV, pT3bN2M0. As the patient developed a metastasis to the skin after radical nephrectomy, we resected the skin metastasis for treatment and specimens were subcutaneously transplanted into SCID mice. Previous studies reported that primary RCC xenografts retained histological or genetic characteristics of the patient tumor, and thus could be a useful treatment model of the corresponding tumors in humans 22. The KURC3 xenograft model mostly recaptured the histopathological features of the original tumors in terms of tumor grade and architecture (Fig. S6A). The *VHL* mutation, p.Phe76del (c.227‐229delTCT), was identified in primary and xenograft tumors. As KURC3 expressed higher levels of CCL2 compared with other types of KURC (Fig. S6B), we used the KURC3 xenograft model for further experiments.

**Figure 5:**
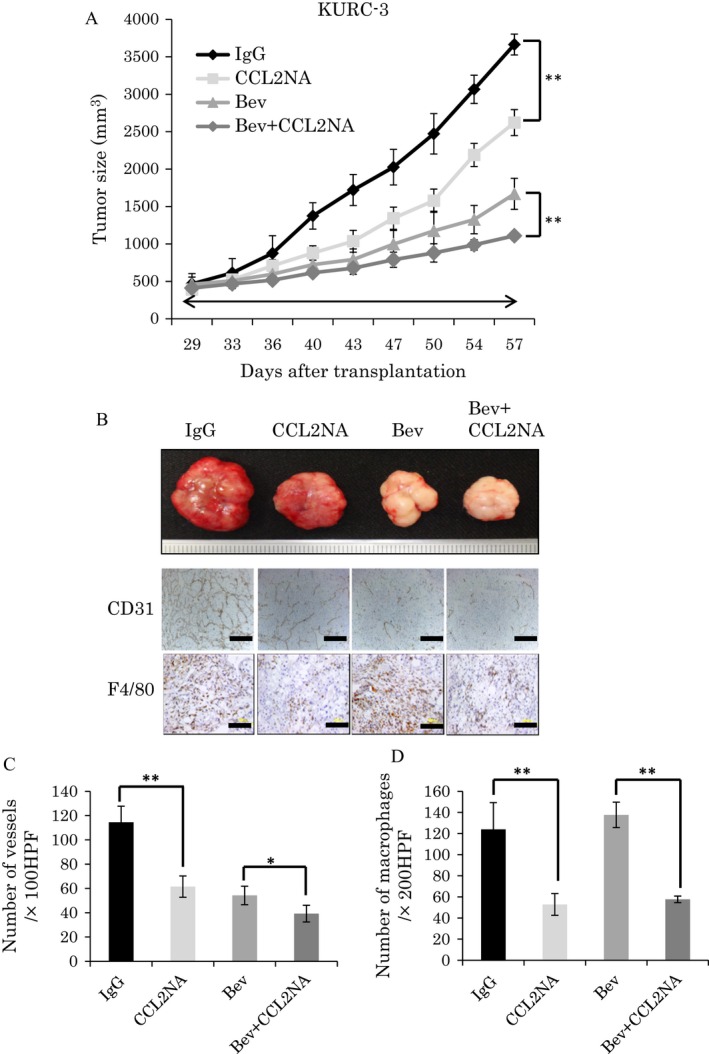
Antitumor effect of CCL2NA and bevacizumab in primary renal cell carcinoma (RCC) xenografts. Primary RCC xenografts were treated with IgG (control), CCL2NA, bevacizumab, or bevacizumab plus CCL2NA (*n* = 4 mice per each group). (A) Tumor growth curve: each line represents the average tumor size of each treatment group. Values represent mean ± SD. Statistical analysis was performed using the two‐way repeated ANOVA (**P <* *0.01). (B) Tumor images (top) and images of CD31 and F4/80 immunohistochemical staining (bottom) of tumor tissues in each treatment group (bar: 100 *μ*m). (C, D) Quantification of microvessel density (MVD) (C) or macrophage infiltration (D) of tumor tissues in each treatment group. Values represent mean ± SD. Statistical analysis was performed using the Student's *t*‐test (* P < 0.05, ** P < 0.01).

KURC3 xenografts were inoculated into SCID mice (four mice/group). After approximately 4 weeks of tumor growth, mice were treated with IgG (control), CCL2NA, bevacizumab, or CCL2NA plus bevacizumab. As with the treatment of bevacizumab, CCL2NA significantly inhibited tumor growth compared with IgG treatment (Fig. 5A and B). Interestingly, the combination therapy of CCL2NA and bevacizumab resulted in significant inhibition of tumor growth compared with bevacizumab alone (Fig. 5A and B). Immunohistochemical studies revealed significant decreases in MVD and macrophage infiltration of CCL2NA‐treated tumors compared with IgG‐treated tumors (Fig. 5B, C, and D). Similar to the results from experiments with the 786‐O xenograft model, MVD was decreased but macrophage infiltration was slightly, but not statistically significantly, increased in tumors treated with bevacizumab alone compared with those with IgG treatment. Upon combined treatment with CCL2NA and bevacizumab, however, macrophage infiltration was decreased compared with bevacizumab alone. Collectively, these results indicated that ccRCC, at least one with CCL2 expression, were sensitive to CCL2 inhibition and that caused additional effects in tumors in addition to antiangiogenesis by VEGF inhibition.

## Discussion

Renal cell carcinoma is usually asymptomatic in its early stages, and thus approximately 30% of patients at diagnosis show locally advanced and/or metastatic RCC 23. Treatment of locally advanced and metastatic RCC is difficult because it shows no or limited responsiveness to conventional anticancer therapies, such as radiation, chemotherapy, and immunotherapy (interferon‐*α* and interleukin‐2). Despite current advances in understanding the molecular biology of RCC, there are still limitations in the effect of molecular target therapies such as multiple tyrosine kinase inhibitors and mTOR inhibitors. Thus, it is necessary to develop new therapeutic targets for RCC.

We previously reported that the pVHL‐atypical PKC‐JunB pathway contributed to promote the cell invasiveness and to enhance angiogenesis in ccRCC in a HIF‐independent manner, and we also showed that CCL2 was one of the downstream effectors of JunB by microarray analysis 6.

Regarding CCL2 expression in ccRCC, microarray data derived from Oncomine showed significantly higher expression of CCL2 in cancer tissue compared with normal kidney parenchyma. However, the role of CCL2 in RCC progression remains unclear. In this study, we first showed that CCL2 expression was correlated with higher clinical stage, worse tumor grade, and poor prognosis in ccRCC patients. Importantly, higher expression of CCL2 was significantly associated with the level of macrophage recruitment in the tumors. Indeed, a previous report showed that high levels of TAMs indicated poor prognosis in RCC 24, and this would be consistent with our observation. In addition to RCC, several studies showed that CCL2 promoted tumor progression in other cancers, including breast and rectal cancer, implicating the therapeutic potential of CCL2 25, 26.

To explore the potential effects of CCL2 in RCC, we performed xenograft experiments and observed significant roles of CCL2 in angiogenesis and macrophage recruitment in tumors. Using a WT8 subclone in which CCL2 was overexpressed and a 786‐O subclone in which CCL2 expression was knocked down, we found that CCL2 overexpression enhanced and CCL2 knockdown suppressed tumor growth, angiogenesis, and macrophage infiltration in vivo. Several reports have demonstrated that CCL2 plays an important role in tumor progression. One of the mechanisms of CCL2 in tumor progression is through its involvement in angiogenesis 7. CCL2 can promote tumor angiogenesis through two mechanisms. First, CCL2 can act directly on endothelial cells to promote angiogenesis 27, 28. A previous study also showed that treatment of immunodeficient mice bearing mammary tumors with anti‐CCL2 antibodies resulted in significant inhibition of lung metastases and increase in survival, and the mechanism was attributed to the direct angiogenic effect of CCL2 29. Second, CCL2 is considered to be an important molecule involved in macrophage infiltration. A recent report showed that tumor malignancy is dependent upon interactions in the tumor microenvironment between tumor cells and stromal cells such as immune cells, fibroblasts, and endothelial cells 30. In particular, macrophages are stromal cells that are known to promote tumor invasion, metastasis, and angiogenesis in various tumors 31, 32. Intratumoral macrophages can produce a series of angiogenic factors, including VEGF, FGF2, and MMP9 33. A number of studies in human malignant tumors have found that a higher level of macrophage infiltration is associated with poor prognosis, and these observations indicate that macrophages may promote tumor progression 34. Indeed, this study showed that depletion of macrophages from RCC xenografts overexpressing CCL2 by administration of clodronate liposomes also suppressed tumor growth and angiogenesis. These findings suggested that CCL2 inhibition exhibits antitumor effects against ccRCCs, at least in part, through the regression of macrophage recruitment into the tumor.

In this study, immunohistochemical identification of TAMs was performed using antibodies against CD68 in human clinical samples or against F4/80 in mouse xenograft tumors, as they has been used as a standard surrogate marker for TAMs. However, several reports have described polarization of activated macrophages (such as M1 and M2) within the tumor microenvironment. Because much evidence has indicated that TAMs have the M2 phenotype and highly express CD163, CD204, or MRC1/CD206 11, 35, 36, these markers are thought to be useful to distinguish TAMs with the M2 phenotype from other M1‐polarized macrophages. To clarify the characteristics of macrophages which recruited by CCL2 in our xenograft models, we have performed the immunohistochemical staining of WT8 CCL2 tumors to evaluate iNOS, MRC1/CD206, and arginase1 expression of macrophages. Indeed, we observed expression of macrophages predominantly with M2 marker MRC1/CD206 and Arginase1 but not with M1 marker iNOS, indicating these macrophages predominantly polarized to M2 phenotype in CCL2 overexpressed xenograft tumors (Fig. S7). This observation is consistent with the previous report that infiltrating macrophages were predominantly polarized to M2 phenotype in ccRCC clinical samples or the report that culture supernatants from RCC cell lines induced polarization of macrophages toward the M2 phenotype 24, 37. At present, it is still unclear whether tumor progression with abundant TAMs could be enhanced or these TAMs could be polarized to M2 phenotype mainly by CCL2 overexpression in ccRCC. These questions must be clarified in future studies by taking comparison of cytokines and angiogenic factors derived from cancer cells or stromal cells into consideration in the experimental design to explore the interaction between cells in tumor microenvironment. To examine the effect of inhibiting CCL2 activity in the clinical relevant RCC model, we administered CCL2 neutralizing antibodies to primary RCC xenografts, which similarly resulted in significant suppression of tumor growth, angiogenesis, and macrophage infiltration. Although CCL2NA alone was effective, the combination therapy of CCL2NA and bevacizumab resulted in significant inhibition of tumor growth compared with each agent alone. Intriguingly, macrophage infiltration was slightly increased by bevacizumab alone treatment compared with IgG treatment. However, simultaneous treatment with CCL2NA and bevacizumab could significantly decrease macrophage infiltration. Because one of the suggested mechanisms of resistance to antiangiogenic therapy includes recruitment of macrophage infiltration 38, the combination or sequential therapy with vascular‐targeted agents and inhibitors for CCL2 axis may be useful for application in clinical practice.

Several studies have indicated that modification in the tumor microenvironment by the CCL2 axis also modulates immunological responses. A recent report showed that TAMs isolated from RCC displayed enhanced 15‐lipoxygenase‐2 (15‐LOX2) pathway activity and produced substantial amounts of CCL2 and IL‐10 that contribute to RCC‐related inflammation, immunosuppression, and malignant progression 39. Macrophages also trigger immune cells, for example, T lymphocytes, and immune checkpoints on infiltrating T lymphocytes are key regulators of immune escape in cancer. The efficacy of immune checkpoint blockade with antibodies that target the programmed cell death protein 1 pathway (PD‐1/PD‐L1) and cytotoxic T lymphocyte‐associated antigen 4 (CTLA‐4) have been reported in a variety of malignancies 40. RCC has also demonstrated durable responses to immune checkpoint inhibition 41. It was recently reported that CCL2 influences the expression of PD‐L1 in polymorphonuclear myeloid‐derived suppressor cells (PMN‐MDSCs) and that CCL2 promotes PMN‐MDSC‐mediated suppression of T cells in colorectal cancer 26. Thus, there is the possibility that blocking CCL2 could augment the effect of immunotherapy in a multifactorial immunologic mechanism.

In conclusion, here we demonstrated that CCL2 plays essential role in ccRCC progression by promoting angiogenesis and recruiting macrophages. Although the exact mechanisms of the CCL2 signaling axis in ccRCC should be further clarified, our findings suggest that CCL2 could be a potential therapeutic target for patients with ccRCC.

## Conflict of Interest

The authors declare no competing financial interests.

## Supporting information


**Figure S1.** Oncomine database analysis of CCL2 expression in ccRCC versus normal kidney.
**Figure S2.** (A) CCL2 expression in WT8 mock or WT8 CCL2 cells by qRT‐PCR (left) and ELISA (right). Values represent mean ± SD. Statistical analysis was performed using the Student's *t*‐test (**P <* *0.01). (B) In vitro cell proliferation of WT8 mock or WT8 CCL2 cells. Values represent mean ± SD. Statistical analysis was performed using the Student's *t*‐test (n.s.: not significant). (C) CCL2 expression in 786‐O scramble or 786‐O shCCL2 cells by qRT‐PCR (left) and ELISA (right). Values represent mean ± SD. Statistical analysis was performed using the Student's *t*‐test (**P <* *0.01). (D) In vitro cell proliferation of 786‐O scramble or 786‐O shCCL2 cells. Values represent mean ± SD. Statistical analysis was performed using the Student's *t*‐test (n.s.: not significant).
**Figure S3.** (A) CCL2 expression in UMRC2 VHL mock or UMRC2 VHL CCL2 cells by qRT‐PCR (top table) and ELISA (bottom table). Values represent mean ± SD. Statistical analysis was performed using the Student's *t*‐test (**P <* *0.01). (B) In vitro cell proliferation of UMRC2 VHL mock or UMRC2 VHL CCL2 cells. Values represent mean ± SD. Statistical analysis was performed using the Student's *t*‐test (n.s.: not significant). (C) Local tumor xenografts were established by subcutaneous injection of UMRC2 VHL mock or UMRC2 VHL CCL2 cells to nude mice (*n *= 4 mice per group), and tumor growth was monitored as described in Materials and Methods. Values represent mean ± SD. Statistical analysis was performed using two‐way repeated ANOVA (**P <* *0.01). (D) Expression of CCL2 was analyzed in tumor tissues from UMRC2 VHL mock or UMRC2 VHL CCL2 cells by ELISA. Values represent mean ± SD. Statistical analysis was performed using the Student's *t*‐test (**P <* *0.01). (E) Immunohistochemical detection of CD31 in tumor tissues from UMRC2 VHL mock or UMRC2 VHL CCL2 cells (top panels, bar: 100 *μ*m) and quantitative analysis of CD31 staining (bottom table). Values represent mean ± SD. Statistical analysis was performed using the Student's *t*‐test (**P <* *0.01). (F) Immunohistochemical detection of F4/80 in tumor tissues from UMRC2 VHL mock or UMRC2 VHL CCL2 cells (top panels, bar: 100 *μ*m) and quantitative analysis of F4/80 staining (bottom table). Values represent mean ± SD. Statistical analysis was performed using the Student's *t*‐test (*P <* *0.05).
**Figure S4.** (A) CCL2 expression in UMRC2 scramble or UMRC2 shCCL2 cells by qRT‐PCR (top table) and ELISA (bottom table). Values represent mean ± SD. Statistical analysis was performed using the Student's *t*‐test (**P <* *0.01). (B) In vitro cell proliferation of UMRC2 scramble or UMRC2 shCCL2 cells. Values represent mean ± SD. Statistical analysis was performed using the Student's *t*‐test (n.s.: not significant). (C) Local tumor xenografts were established by subcutaneous injection of UMRC2 scramble or UMRC2 shCCL2 cells to nude mice (*n* = 4 mice per group), and tumor growth was monitored as described in Materials and Methods. Values represent mean ± SD. Statistical analysis was performed using two‐way repeated ANOVA (**P <* *0.01). (D) Expression of CCL2 was analyzed in tumor tissues from UMRC2 scramble or UMRC2 shCCL2 cells by ELISA. Values represent mean ± SD. Statistical analysis was performed using the Student's *t*‐test (**P <* *0.01). (E) Immunohistochemical detection of CD31 in tumor tissues from UMRC2 scramble or UMRC2 shCCL2 cells (top panels, bar: 100 *μ*m) and quantitative analysis of CD31 staining (bottom table). Values represent mean ± SD. Statistical analysis was performed using the Student's *t*‐test (**P <* *0.01). (F) Immunohistochemical detection of F4/80 in tumor tissues from UMRC2 scramble or UMRC2 shCCL2 cells (top panels, bar: 100 *μ*m) and quantitative analysis of F4/80 staining (bottom table). Values represent mean ± SD. Statistical analysis was performed using the Student's *t*‐test (**P <* *0.01).
**Figure S5.** The effect of inhibiting CCL2 activity with a CCL2 neutralizing antibody (CCL2NA) in xenograft tumors derived from CCL2‐secreting 786‐O cells. (A) Tumor growth curve: the average tumor size of xenografts treated with IgG (control) or CCL2NA (*n *= 4 mice per each group). Values represent mean ± SD. Statistical analysis was performed using two‐way repeated ANOVA (**P <* *0.01). (B) Tumor growth curve: the average tumor size of xenografts treated with IgG (control) or bevacizumab (*n* = 4 mice per each group). Values represent mean ± SD. Statistical analysis was performed using two‐way repeated ANOVA (**P <* *0.01). (C) Images of CD31 and F4/80 immunohistochemical staining of tumor tissues from 786‐O cells treated with IgG (control), CCL2NA, or bevacizumab. (D, E) Quantification of MVD (D) or macrophage infiltration (E) in tumor tissues from 786‐O cells treated with IgG (control), CCL2NA, or bevacizumab. Values represent mean ± SD. Statistical analysis was performed using the Student's *t*‐test (**P <* *0.01).
**Figure S6.** H&E staining of an original RCC surgical specimen, skin metastasis specimen, and xenograft KURC3 tumor tissue (bar: 100 *μ*m). Expression of CCL2 was analyzed in tumor tissues of KURC1 or KURC3 by ELISA.
**Figure S7.** Immunohistochemical detection of F4/80, MRC1/CD206, arginase1, and iNOS with infiltrating macrophage (arrows) in tumor tissues derived from WT8 CCL2 cells (bar: 100 *μ*m).
