# Supplementary material for: CCL2 as a potential therapeutic target for clear cell renal cell carcinoma
Source: Cancer Med. 2016 Sep 26;5(10):2920–33. doi: 10.1002/cam4.886 (PMC5083746; doi:10.1002/cam4.886)

Figure S1

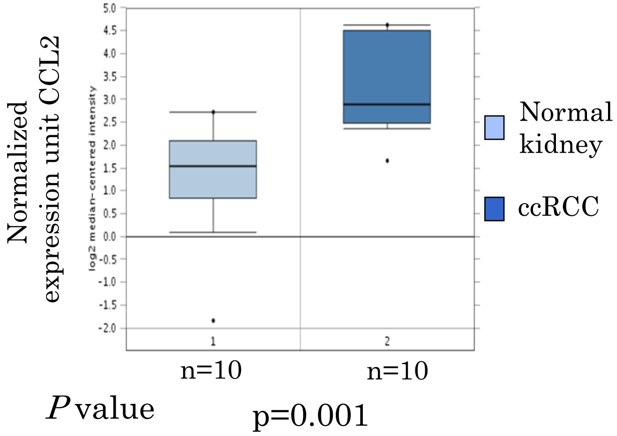

Gumz Renal Statistics

Figure S2

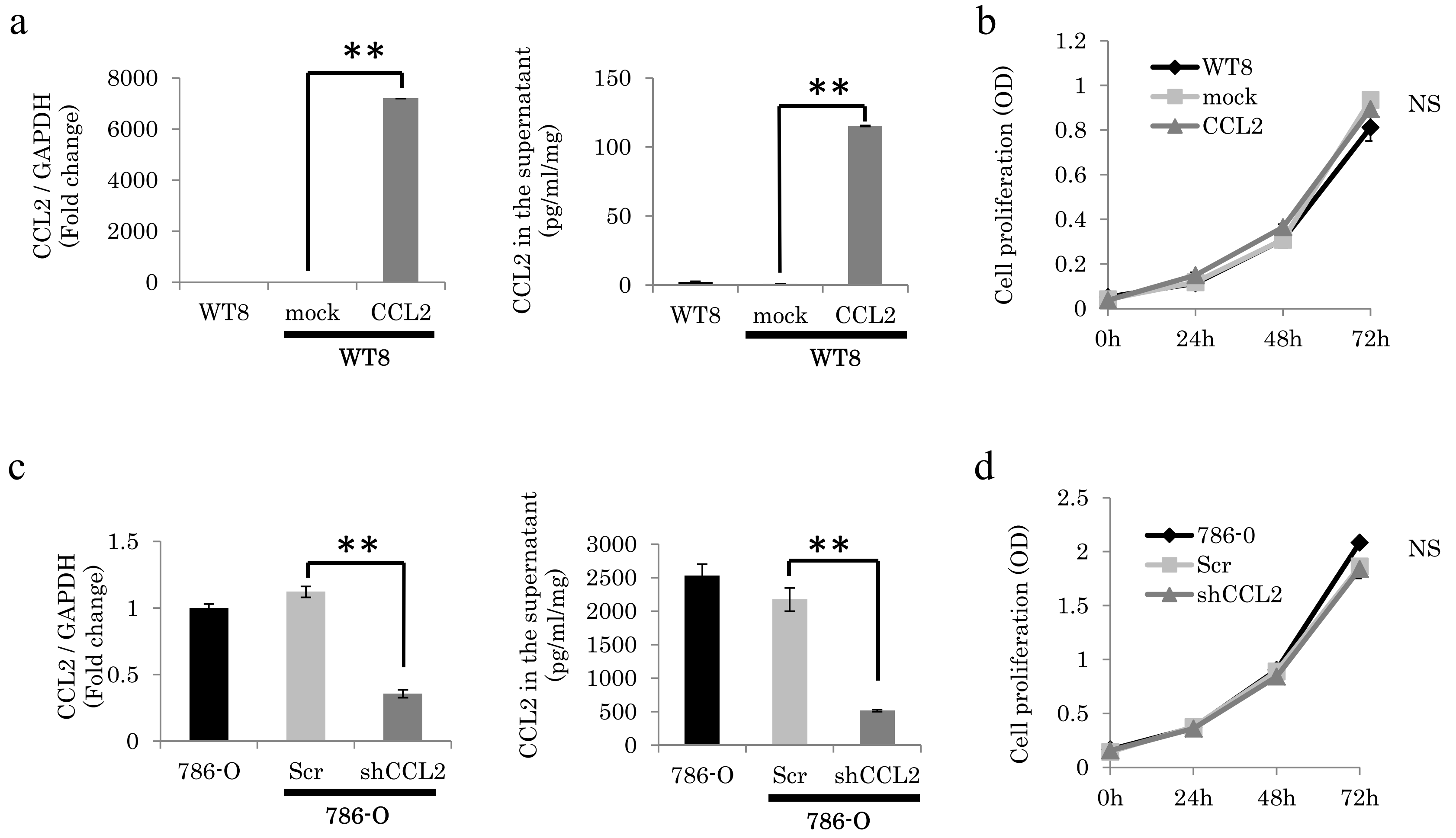

a
8000
6000
4000
2000
0
CCL2 / GAPDH
(Fold change)
**
WT8
mock
CCL2
WT8
150
100
50
0
CCL2 in the supernatant
(pg/ml/mg)
**
WT8
mock
CCL2
WT8
b
1.2
1
0.8
0.6
0.4
0.2
0
Cell proliferation (OD)
WT8
mock
CCL2
NS
0h
24h
48h
72h
c
1.5
1
0.5
0
CCL2 / GAPDH
(Fold change)
**
786-O
Scr
shCCL2
786-O
3000
2500
2000
1500
1000
500
0
CCL2 in the supernatant
(pg/ml/mg)
**
786-O
Scr
shCCL2
786-O
d
2.5
2
1.5
1
0.5
0
Cell proliferation (OD)
786-0
Scr
shCCL2
NS
0h
24h
48h
72h

Figure S3

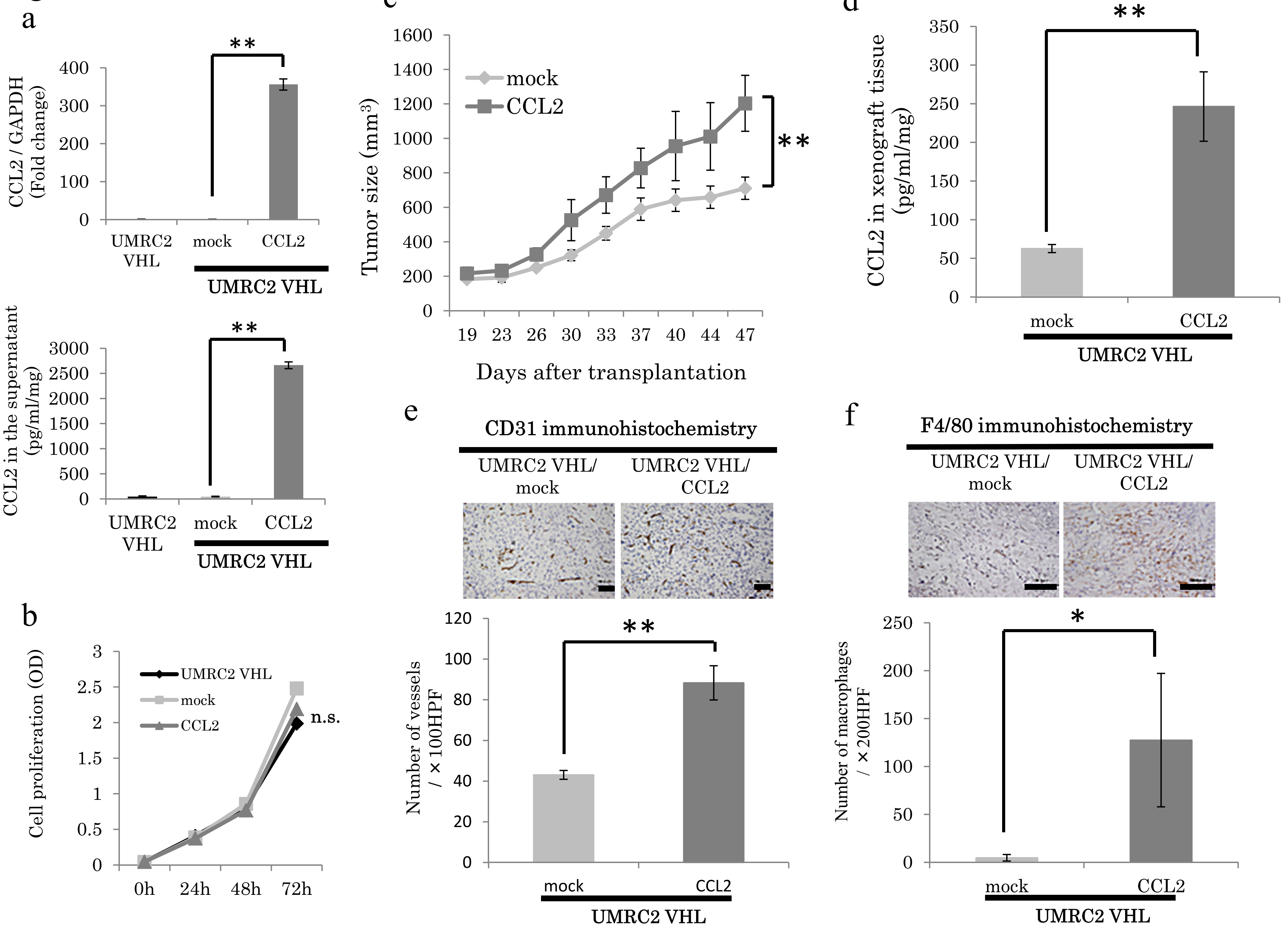

a
CCL2 / GAPDH (Fold change)
400
300
200
100
0
UMRC2 VHL
mock
CCL2
UMRC2 VHL
**
CCL2 in the supernatant (pg/ml/mg)
3000
2500
2000
1500
1000
500
0
UMRC2 VHL
mock
CCL2
UMRC2 VHL
**
b
Cell proliferation (OD)
UMRC2 VHL
mock
CCL2
n.s.
0h
24h
48h
72h
c
Tumor size (mm3)
mock
CCL2
**
19 23 26 30 33 37 40 44 47
Days after transplantation
d
CCL2 in xenograft tissue (pg/ml/mg)
**
mock
CCL2
UMRC2 VHL
e
CD31 immunohistochemistry
UMRC2 VHL/ mock
UMRC2 VHL/ CCL2
Number of vessels / × 100HPF
**
mock
CCL2
UMRC2 VHL
f
F4/80 immunohistochemistry
UMRC2 VHL/ mock
UMRC2 VHL/ CCL2
Number of macrophages / × 200HPF
*
mock
CCL2
UMRC2 VHL

Figure S4

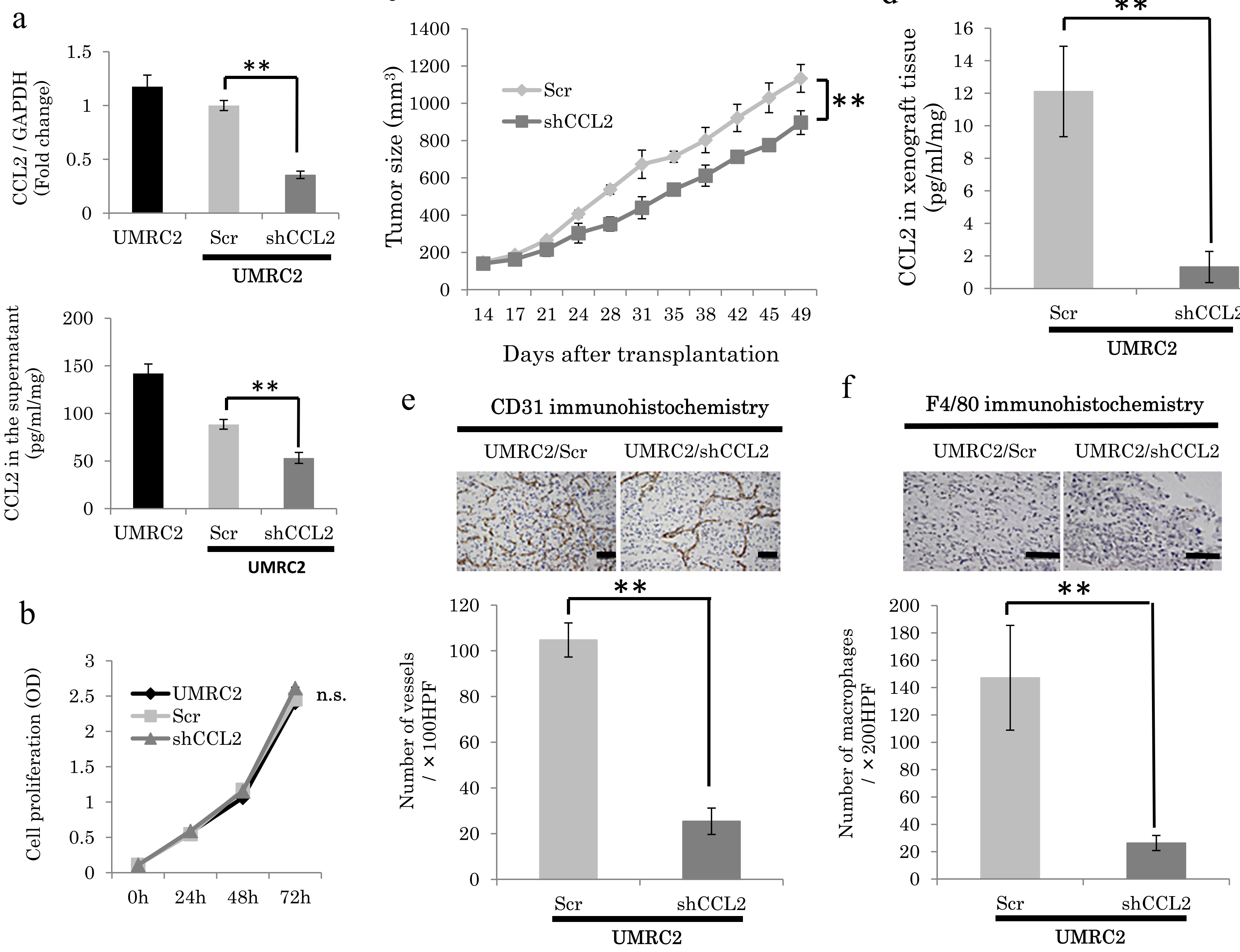

a
CCL2 / GAPDH (Fold change)
1.5
1
0.5
0
UMRC2
Scr
shCCL2
UMRC2
**
CCL2 in the supernatant (pg/ml/mg)
200
150
100
50
0
UMRC2
Scr
shCCL2
UMRC2
**
b
Cell proliferation (OD)
3
2.5
2
1.5
1
0.5
0
UMRC2
Scr
shCCL2
n.s.
0h
24h
48h
72h
c
Tumor size (mm3)
1400
1200
1000
800
600
400
200
0
Scr
shCCL2
**
14
17
21
24
28
31
35
38
42
45
49
Days after transplantation
d
CCL2 in xenograft tissue (pg/ml/mg)
16
14
12
10
8
6
4
2
0
**
Scr
shCCL2
UMRC2
e
CD31 immunohistochemistry
UMRC2/Scr
UMRC2/shCCL2
Number of vessels / ×100HPF
120
100
80
60
40
20
0
**
Scr
shCCL2
UMRC2
f
F4/80 immunohistochemistry
UMRC2/Scr
UMRC2/shCCL2
Number of macrophages / ×200HPF
200
180
160
140
120
100
80
60
40
20
0
**
Scr
shCCL2
UMRC2

Figure S5

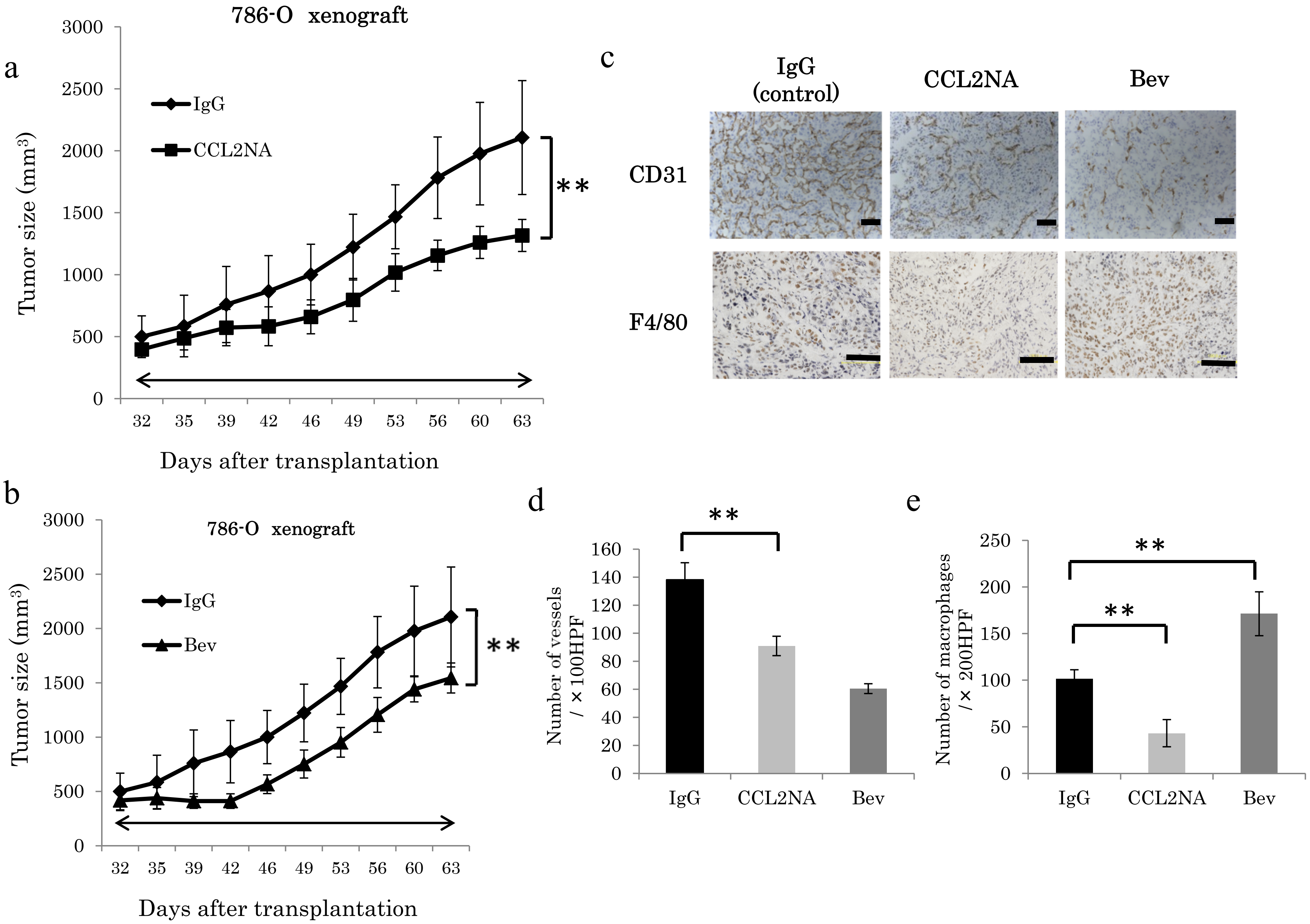

a
786-O xenograft
Tumor size (mm3)
IgG
CCL2NA
**
Days after transplantation
b
786-O xenograft
IgG
Bev
**
c
IgG (control)
CCL2NA
Bev
CD31
F4/80
d
Number of vessels / ×100HPF
**
IgG
CCL2NA
Bev
e
Number of macrophages /× 200HPF
**
**
IgG
CCL2NA
Bev

Figure S6

a

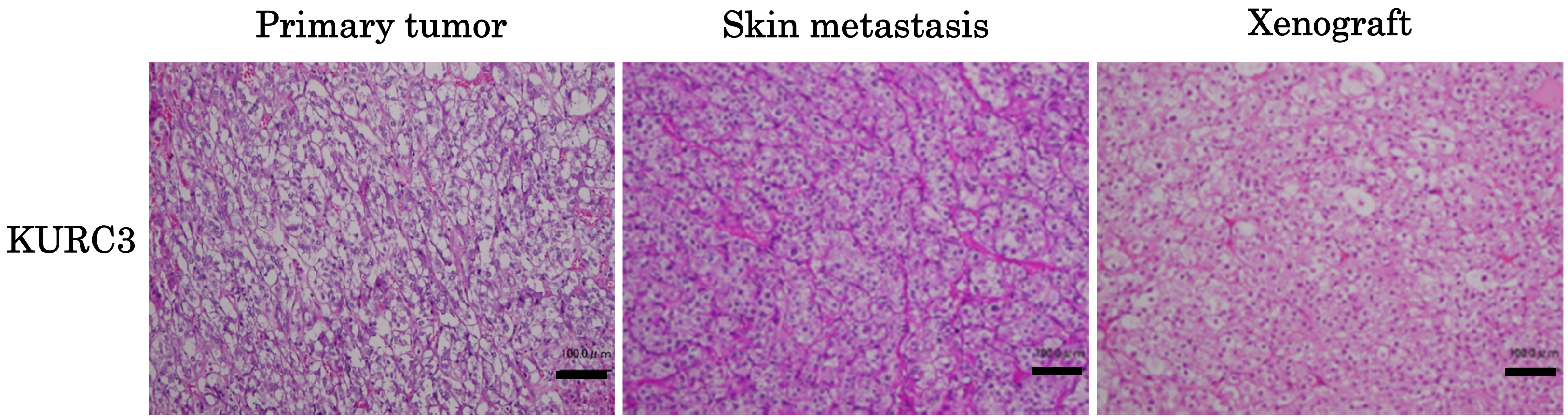
Primary tumor
Skin metastasis
Xenograft
KURC3

b

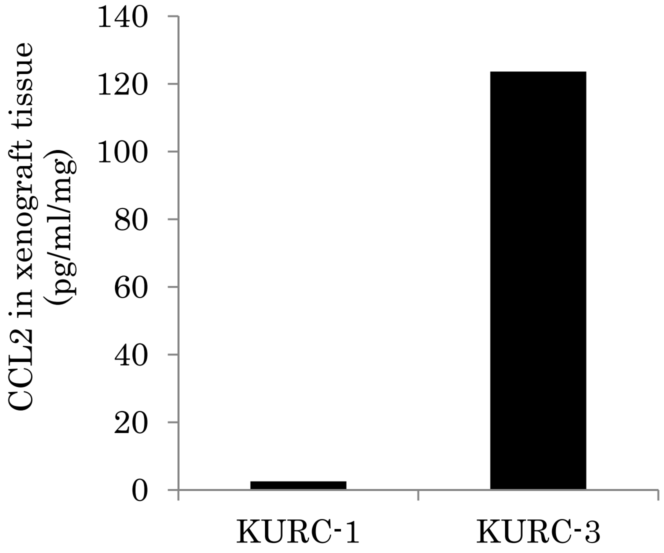
CCL2 in xenograft tissue
(pg/ml/mg)
140
120
100
80
60
40
20
0
KURC-1
KURC-3

Figure S7

WT8 CCL2 xenograft

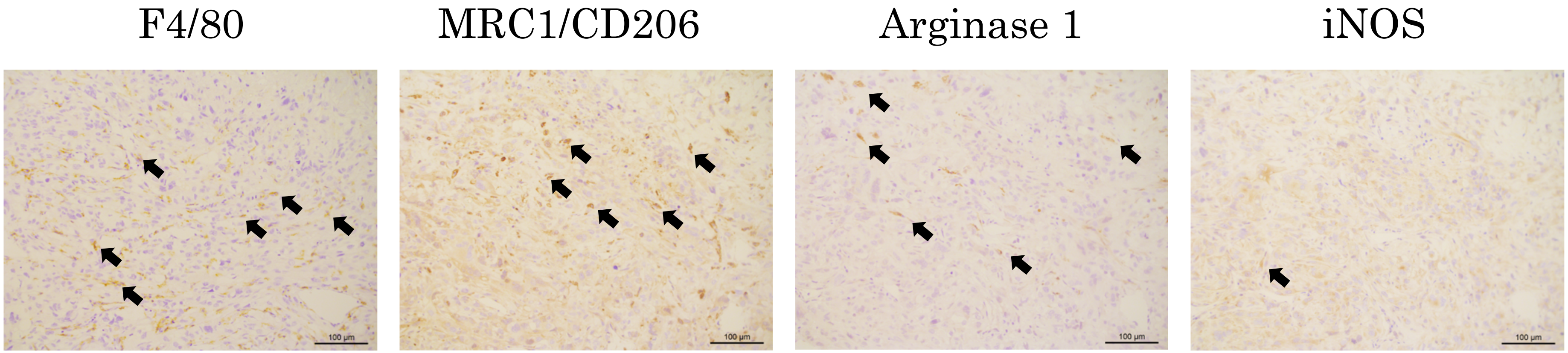

Supplement: Supplementary file 1 — Figure S1. Oncomine database analysis of CCL2 expression in ccRCC versus normal kidney. Figure S2. (A) CCL2 expression in WT8 mock or WT8 CCL2 cells by qRT‐PCR (left) and ELISA (right). Values represent mean ± SD. Statistical analysis was performed using the Student's t‐test (**P < 0.01). (B) In vitro cell proliferation of WT8 mock or WT8 CCL2 cells. Values represent mean ± SD. Statistical analysis was performed using the Student's t‐test (n.s.: not significant). (C) CCL2 expression in 786‐O scramble or 786‐O shCCL2 cells by qRT‐PCR (left) and ELISA (right). Values represent mean ± SD. Statistical analysis was performed using the Student's t‐test (**P < 0.01). (D) In vitro cell proliferation of 786‐O scramble or 786‐O shCCL2 cells. Values represent mean ± SD. Statistical analysis was performed using the Student's t‐test (n.s.: not significant). Figure S3. (A) CCL2 expression in UMRC2 VHL mock or UMRC2 VHL CCL2 cells by qRT‐PCR (top table) and ELISA (bottom table). Values represent mean ± SD. Statistical analysis was performed using the Student's t‐test (**P < 0.01). (B) In vitro cell proliferation of UMRC2 VHL mock or UMRC2 VHL CCL2 cells. Values represent mean ± SD. Statistical analysis was performed using the Student's t‐test (n.s.: not significant). (C) Local tumor xenografts were established by subcutaneous injection of UMRC2 VHL mock or UMRC2 VHL CCL2 cells to nude mice (n = 4 mice per group), and tumor growth was monitored as described in Materials and Methods. Values represent mean ± SD. Statistical analysis was performed using two‐way repeated ANOVA (**P < 0.01). (D) Expression of CCL2 was analyzed in tumor tissues from UMRC2 VHL mock or UMRC2 VHL CCL2 cells by ELISA. Values represent mean ± SD. Statistical analysis was performed using the Student's t‐test (**P < 0.01). (E) Immunohistochemical detection of CD31 in tumor tissues from UMRC2 VHL mock or UMRC2 VHL CCL2 cells (top panels, bar: 100 μm) and quantitative analysis of CD31 staining (bottom table). Val [file CAM4-5-2920-s001.pdf]
